# Healthspan Enhancement by Olive Polyphenols in *C. elegans* Wild Type and Parkinson’s Models

**DOI:** 10.3390/ijms21113893

**Published:** 2020-05-29

**Authors:** Gabriele Di Rosa, Giovanni Brunetti, Maria Scuto, Angela Trovato Salinaro, Edward J. Calabrese, Roberto Crea, Christian Schmitz-Linneweber, Vittorio Calabrese, Nadine Saul

**Affiliations:** 1 Department of Biomedical and Biotechnological Sciences, University of Catania, 95125 Catania, Italy; dirosagabriele85@gmail.com (G.D.R.); q.burneti@gmail.com (G.B.); mary-amir@hotmail.it (M.S.); trovato@unict.it (A.T.S.); 2 Department of Environmental Health Sciences, Morrill I, N344, University of Massachusetts, Amherst, MA 01003, USA; edwardc@schoolph.umass.edu; 3 Oliphenol LLC., 26225 Eden Landing Road, Unit C, Hayward, CA 94545, USA; robertocrea48@gmail.com; 4 Faculty of Life Sciences, Institute of Biology, Molecular Genetics Group, Humboldt University of Berlin, Philippstr. 13, House 22, 10115 Berlin, Germany; christian.schmitz-linneweber@rz.hu-berlin.de (C.S.-L.); nadine.saul@gmx.de (N.S.)

**Keywords:** *C. elegans*, polyphenols, olive oil, healthspan, lifespan, ageing, Parkinson’s disease

## Abstract

Parkinson’s disease (PD) is the second most prevalent late-age onset neurodegenerative disorder, affecting 1% of the population after the age of about 60 years old and 4% of those over 80 years old, causing motor impairments and cognitive dysfunction. Increasing evidence indicates that Mediterranean diet (MD) exerts beneficial effects in maintaining health, especially during ageing and by the prevention of neurodegenerative disorders. In this regard, olive oil and its biophenolic constituents like hydroxytyrosol (HT) have received growing attention in the past years. Thus, in the current study we test the health-promoting effects of two hydroxytyrosol preparations, pure HT and Hidrox^®^ (HD), which is hydroxytyrosol in its “natural” environment, in the established invertebrate model organism *Caenorhabditis elegans*. HD exposure led to much stronger beneficial locomotion effects in wild type worms compared to HT in the same concentration. Consistent to this finding, in OW13 worms, a PD-model characterized by α-synuclein expression in muscles, HD exhibited a significant higher effect on α-synuclein accumulation and swim performance than HT, an effect partly confirmed also in swim assays with the UA44 strain, which features α-synuclein expression in DA-neurons. Interestingly, beneficial effects of HD and HT treatment with similar strength were detected in the lifespan and autofluorescence of wild-type nematodes, in the neuronal health of UA44 worms as well as in the locomotion of rotenone-induced PD-model. Thus, the hypothesis that HD features higher healthspan-promoting abilities than HT was at least partly confirmed. Our study demonstrates that HD polyphenolic extract treatment has the potential to partly prevent or even treat ageing-related neurodegenerative diseases and ageing itself. Future investigations including mammalian models and human clinical trials are needed to uncover the full potential of these olive compounds.

## 1. Introduction

Emerging research has recently focused on increasing the life expectancy of humans which is, however, accompanied by a progressively greater prevalence of neurodegenerative disorders, notably Parkinson’s disease (PD). PD is the second most prevalent late-age onset neurodegenerative disorder affecting 1% of the population after the age of about 60 years old and 4% of those over 80 years old, causing motor impairments, cognitive dysfunction, sleep difficulties, autonomic dysfunctions, and pain [1]. PD is characterized by the progressive loss of dopaminergic neurons in the substantia nigra pars compacta (*SNpc*) of the midbrain area [2]. At the cellular level, the neuropathological hallmarks of PD include intra-cytoplasmic inclusions that contain α-synuclein aggregates, a primary component of intraneuronal Lewy bodies and Lewy neurites in vulnerable neurons of the brain [3]. The loss of dopaminergic neurons results in major motor impairments including resting tremor, muscle rigidity, bradykinesia and postural instability. Since PD affects neurons in the central and peripheral nervous systems, patients typically also exhibit multiple non-motor symptoms including anxiety, depression, memory loss, perturbed proteostasis, mitochondrial dysfunction, oxidative stress, dysregulation of redox homeostasis as well as neurotoxicity [4]. Although the etiology of PD is currently unknown, genetic and environmental triggers are two major factors that play a role in the development of the disease, with the environment accounting for over two-thirds of all cases [5]. Recently, longitudinal studies have identified at least 23 loci and 19 disease-causing genes for familial parkinsonism associated with the progression of non-motor symptoms in PD patients [6,7]. Moreover, several studies have suggested that mitochondrial complex I deficiency in different brain regions is associated with impairment of energy metabolism and neuronal death in PD [8]. It has been postulated that neuroinflammatory processes might play a crucial role in the pathogenesis of PD. The proteomic approach revealed accumulation of neurotoxic misfolded α-synuclein aggregates inducing microglial activation associated to loss of dopaminergic neurons in nigrostriatal system underlying PD pathogenesis [9]. Although treatments are available to alleviate motor symptoms, currently, there are no preventive therapies that can target and lessen PD progression. Recently, epidemiological and clinical studies have supported the idea that Mediterranean diet (MD) is strongly associated with lifespan extension as well as with healthy aging process by reducing the progression of age-related pathologies [10]. The beneficial effects of natural polyphenols and derivatives comprise multi-target activities including the anti-amyloid aggregation, antioxidant, antimicrobial, antihypertensive, hypoglycemic, antiproliferative and vasodilator effects, as well as redox homeostasis activities through a direct modulation of enzymes and proteins involved in stress response pathways [11,12,13,14]. Particular attention has been paid recently by our laboratory to the effects of natural olive polyphenols such as hydroxytyrosol (HT) and oleuropein aglycone (OLE), [10,15] known to possess healthspan benefits against α-synuclein aggregation into intracellular Lewy bodies, as found in PD neurons of the mesencephalic substantia nigra [16]. In addition, in vitro and in vivo studies have shown that HT exerts various protective effects, particularly, strong anti-oxidant and radical scavenging activities [17,18]. With regard to mechanism of polyphenol action, the biological concept of hormesis has emerged as a significant dose response model in the field of neuroprotection elicited by low dose of olive polyphenols [19]. Notably, increasing evidence suggests that mild stressors such as HT may offer beneficial effects in a hormetic-like manner by activating Nrf2-stress response pathway and enhancing brain resilience, neuroplasticity as well as lifespan in vitro and in experimental PD models [20,21]. Moreover, HT activates the Nrf2–antioxidant response element (ARE) pathway, leading to the activation of phase II detoxifying enzymes and the protection of dopaminergic neurons exposed to hydrogen peroxide or to 6-hydroxydopamine [22,23]. This is consistent with the idea that neurohormesis may have anti-aging effects due to induction of adaptive pathways triggered to cope with a mild neuronal stress and open novel potential therapeutic strategies for clinical interventions against the onset and/or progression of PD in humans. In these ways, neurohormetic polyphenols might protect neurons against injury and disease by stimulating the production of antioxidant enzymes, neurotrophic factors, protein chaperones and other proteins that help cells to withstand stress [24,25]. Interestingly, our recent in vivo study with olive polyphenols has demonstrated that HT and OLE exert neuroprotective effects, an improved overall healthspan and, in part, longevity in *Caenorhabditis elegans* (*C. elegans*) models of PD and wild type [15]. 

The nematode *C. elegans* is a multicellular model organism that offers several advantages for investigating both aging and neurodegenerative disorders [26]. The biological features of *C. elegans* are multiple and including short life cycle (i.e., about 3.5 days from egg to adult) and a lifespan of only about 20 days, transparent body, conserved gene network and neurological pathways [27,28]. Moreover, comparative proteomics indicates that for 83% of the *C. elegans* proteome human homologous can be identified [29]. Comparative genomic analysis also shows that nearly 53% of the human protein-coding genome has recognizable worm orthologues [30]. Furthermore, there is a tight connection between lifespan extension and resistance to diverse environments [31]. In this regard, several studies indicate that different stressors acting in hormetic-like manner extend lifespan in *C. elegans* [32], and suggest that hormetic effects could be exploited to prevent the onset of neurodegenerative diseases [20]. Most importantly, its complete genomic sequence is available. Therefore, *C. elegans* as a model organism is widely applied for screening natural bioactive compounds [33]. Several polyphenols effectively increase healthspan and lifespan as well as mitochondrial function in *C. elegans* [34,35,36,37,38]. Notably, *C. elegans* represents an excellent model to study the neuroprotective effects of olive polyphenols. In this context, recent research has demonstrated that extracts from olive leaves efficiently scavenged free radicals in vitro and significantly increased the expression of antioxidant enzymes extending lifespan and increased stress resistance in *C. elegans* [39,40]. 

In the current study we focus on the health-promoting effects of two hydroxytyrosol preparations, pure hydroxytyrosol and Hidrox^®^. Hidrox^®^/ Olivenol Plus™ (HD) is a patented freeze-dried hydroxytyrosol-rich formulation obtained from the acidic hydrolysis of olive vegetation water (OVW or olive juice) and where hydroxytyrosol (40–45% at the total water-soluble olive polyphenols) is maintained in its “natural” environment [41]. Olive juice (aqueous fraction) represents 50% of the weight of the olive fruit and is normally discarded as wastewater. Several findings have reported that HD displays different activity than pure or synthetic HT [40]. International in vitro and in vivo studies showed the health benefits and efficacy of HD as anti-inflammatory, anti-oxidant, anti-scavenger as well as anti-aggregating compound, particularly in PD [42]. 

We hypothesized that the polyphenolic treatments, Hidrox^®^ and pure hydroxytyrosol have the capacity to increase the mean lifespan of *C. elegans* in the presence and absence of thermal stress. Furthermore, it is assumed that they are able to counteract the age-related deterioration of general health parameters, which were assessed by determining the swim performance as a measure of overall body fitness as well as the autofluorescence as one of the most popular ageing biomarkers [43]. Moreover, numerous in vitro studies were already successfully performed to verify the anti-PD effects of olive ingredients [14], however, in vivo studies were rarely conducted. Therefore, by using one chemically-induced and two transgenic PD models of *C. elegans*, the polyphenolic treatments were tested for their anti-PD effects in vivo. Besides the swim performance, neuronal degeneration as well as α-synuclein accumulation were taken into account to assess the anti-PD potential. Finally, it was hypothesized that HD is even more effective in preventing PD- and ageing-related symptoms than HT, which was tested by a direct comparison of the action of HT and HD. 

## 2. Results

### 2.1. HD and HT Enhanced the Health and Lifespan of Wild Type Nematodes

As stress resistance is one of the key features characterizing the health status of an organism [44], heat stress resistance was determined in the presence and absence of HD in different concentrations. This test was also used to find the optimal concentration for further investigations. The results of HT treatment are shown in addition to enable the direct comparison between pure and mixed polyphenol treatments.

**Figure 1:**
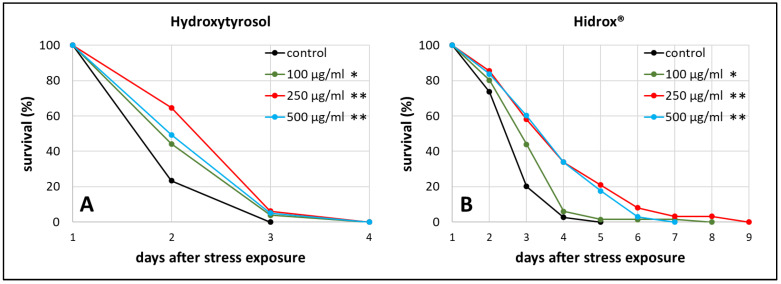
Heat stress resistance in the presence of HT and HD. At the third day of adulthood (day 1) wild type nematodes were exposed to heat stress at 37 °C for 3 h prior monitoring survival. The survival is plotted as the percentage of the initial population in the control group as well as in the HT (**A**) and HD (**B**) treated groups. Three biological replicates were combined with a total of ≥52 nematodes per treatment. Statistical significance was calculated by log-rank test. Differences compared to control were considered significant at *p* < 0.05 (*) and *p* < 0.001 (**).

We observed the survival of worms starting from the 3rd day of adulthood, which was the day of heat stress exposure, until all worms died. The mean survival, which refers to the time between that stress exposure day until the end of the test, was increased by about 22% (from 2.23 days in the control group to 2.71 days in the treated group) during 250 µg/mL HT treatment (Figure 1A), whereas 250 µg/mL HD extended the survival by even 40% (from 2.96 in the control to 4.13 days) (Figure 1B). Moreover, the maximum survival, defined as the time point when 90% of the population was dead, increased by 63% during 250 µg/mL HD treatment (*p* ≤ 0.001 with Fisher’s Exact Test) and only by 14% during 250 µg/mL HT treatment (*p* ≤ 0.05 with Fisher’s Exact Test) compared to the respective control. 250 µg/mL was the most effective concentration for both treatments in terms of mean and maximum survival after stress exposure (Table S1), thus, this concentration was selected for the following experiments.

**Figure 2:**
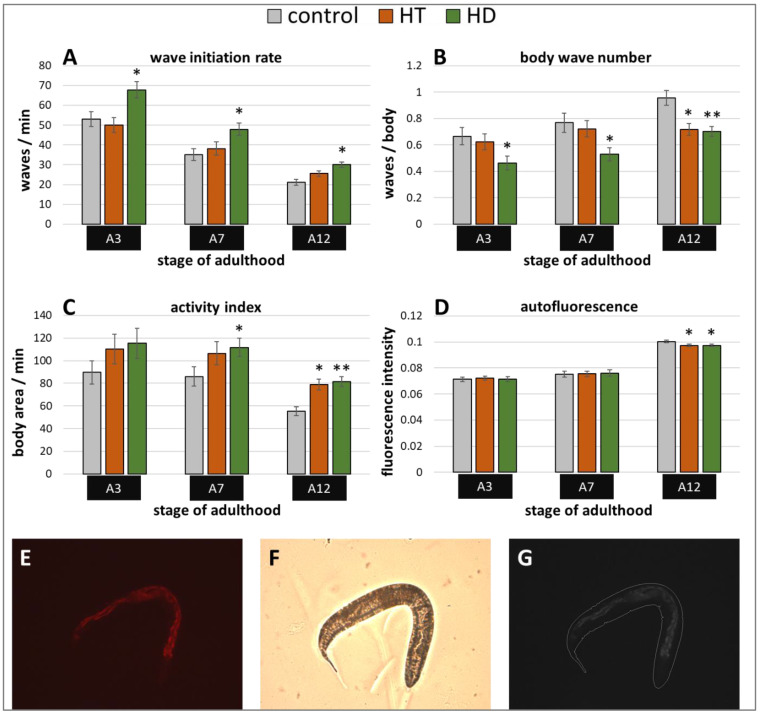
Healthspan benefits in wild type nematodes treated with HD and HT. The analysis of locomotion after polyphenol treatments comprises three parameters: the wave initiation rate (**A**), the body wave number (**B**) and the activity index (**C**). In two independent repeats, a total of ≥63 nematodes were analysed per treatment and age. In addition, the autofluorescence was monitored in two biological repeats with a total of ≥37 nematodes per treatment and age (**D**). Data are represented as mean ± SEM and statistical differences compared to control were considered significant at *p* < 0.05 (*) and *p* < 0.001 (**). A3, A7, A12: 3rd, 7th and 12th day of adulthood. Finally, an example for the typical appearance of red autofluorescence is shown (**E**) with the respective shot in bright field (**F**) as well as the merged and processed picture for the analysis in CellProfiler (**G**).

Besides stress resistance, locomotion is one of the most important features that reflects the general fitness and health status of nematodes [45,46] and shows a constant decline during the ageing process [47]. Therefore, the swimming behavior was monitored in three different age classes with and without polyphenol treatment. Three age-dependent motion-parameters were chosen, that is wave initiation rate (often referred to as thrashing speed), activity index, and body wave number. The wave initiation rate is the number of body waves per minute, which indicates the movement-speed, whereas the activity index adds up the number of pixels that are covered by the nematode during the time spent for two strokes as an indicator for the vigorousness of bending over time. Furthermore, the body wave number, which is low in healthy and young worms, determines the waviness of the body at each time point. The data obtained in the current study verify the age-dependence of all selected swim parameters (Figure 2A–C). As previously described in Restif et al. [47] and Ibáñez-Ventoso et al. [48], the wave initiation rate (Figure 2A) and the activity index (Figure 2C) decreased with age, whereas the body wave number (Figure 2B) increased during ageing.

Interestingly, the movement speed was not influenced by 250 µg/mL HT in any age group, but 250 µg/mL HD could provoke an increase of 28%, 36%, and 42% in the wave initiation rate at the 3rd, 7th, and 12th day of adulthood, respectively (Figure 2A). Furthermore, HD was also able to enhance healthspan by decreasing the body wave number by at least 27% in all three age groups (Figure 2B) and by increasing the activity index by 30% and 48% at the 7th and 12th day of adulthood (Figure 2C), whereas HT did only positively influence these parameters at the 12th day of adulthood.

In addition, a well-known biomarker was investigated to analyse the beneficial effects of 250 µg/mL HD on the healthspan of *C. elegans*. The amount of autofluorescent material, sometimes referred to as lipofuscin or “age pigment”, increases in *C. elegans* during ageing [49]. It was shown that red autofluorescence, which is mainly located in the intestine, reflects the ageing and health status of nematodes in the most reliable way [43]. Therefore, red fluorescence was measured in *C. elegans* during ageing, whereas the total intensity was calculated per worm body as illustrated by Figure 2E–G. Both treatments, HT and HD, were able to decrease the accumulation of the fluorescent material at the 12th day of adulthood by 3%, an effect which was absent in younger worms (Figure 2D).

**Figure 3:**
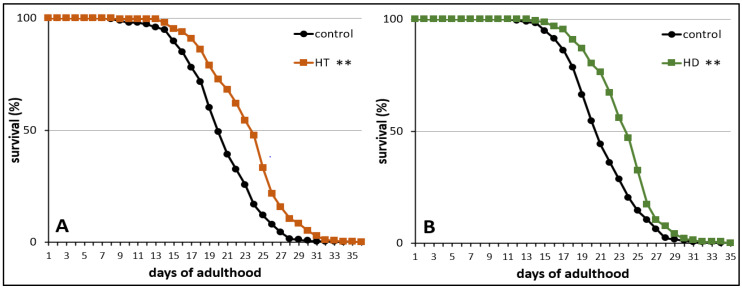
Life prolonging effects of HT and HD in wild type. The survival curves of control and polyphenol treated nematodes are shown. Survival is expressed as a percentage of the initial population per day. The curves represent three independent trials with a total of 250 and 286 nematodes in the control and HT treated nematodes, respectively (**A**) and two independent trials with a total of 172 and 144 nematodes in the control and HD treated nematodes, respectively (**B**). Statistical significance was calculated by log-rank test; differences compared to control were considered significant at *p* < 0.001 (**).

Finally, the influence of HT and HD on the mortality rate was measured in wild type nematodes under standard laboratory conditions. The treatment with 250 mg/mL HT and HD resulted in an increase of mean lifespan by 14% (Figure 3A) and 12% (Figure 3B), respectively. The maximum lifespan, however, was only slightly (without significance according to the Fisher’s Exact Test) increased by 7% after HD-treatment and the biggest increase was visible in the median lifespan (16%). A similar pattern was observed in the HT-treated group.

### 2.2. Rotenone-Induced Parkinsonian Models in C. elegans Profit from HT and HD

**Figure 4:**
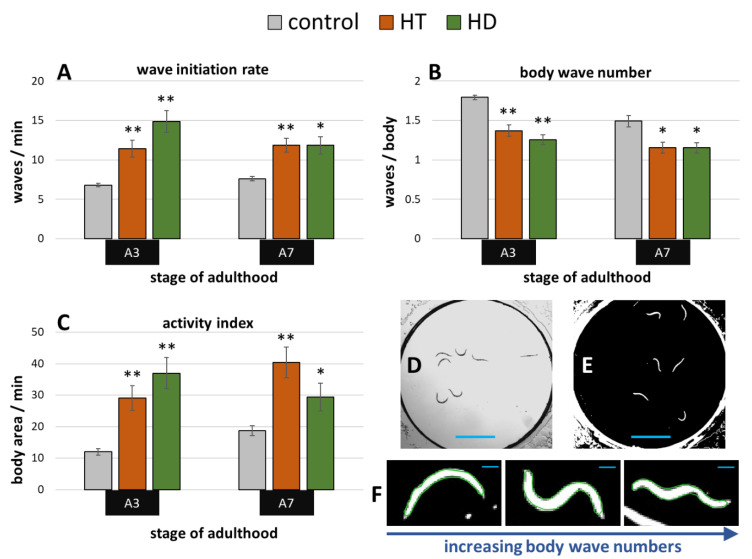
Effect of HD and HT on rotenone induced locomotion impairment. 10 µM rotenone administration with and without simultaneous HD or HT treatment started at the fourth larval stage. The wave initiation rate (**A**), the body wave number (**B**) and the activity index (**C**) are shown at the third (A3) and seventh (A7) day of adulthood. Data are pooled from two biological repeats with ≥44 worms per treatment and age. The bars represent the mean ± SEM and differences compared to control were considered significant at *p* < 0.05 (*) and *p* < 0.001 (**). The appearance of single frames in swim analysis are shown before (**D**) and after (**E**) image processing. In addition, single nematodes recognized by CeleST (as indicated by the green outline) are shown with increasing number of body waves (**F**). The blue scale bars represent 2.5 mm (**D**,**E**) and 200 µm (**F**), respectively.

Exposure to the pesticide rotenone induces the parkinsonian syndrome in wild type *C. elegans*, which manifests in impaired fitness and movement [50]. A swim assay (illustrated in Figure 4D,E) was performed to determine whether HD and HT are able to reduce the rotenone induced symptoms. 10 µM rotenone strongly impaired the movement capacities at the 3rd and 7th day of adulthood in all tested parameters (Figure 4A–C) when compared to untreated nematodes at the same age (Figure 2A–C). Interestingly, both polyphenol treatments showed similar beneficial effects on nematodes suffering from rotenone at both tested ages. The body wave number (illustrated in Figure 4F) was decreased by up to 30% and 24% after HD- and HT-treatment, respectively (Figure 4B). The thrashing speed was increased after HT-treatment by 68% and 56% at the 3rd and 7th day of adulthood, respectively and by 119% and 55% at the 3rd and 7th day of adulthood, respectively, after HD- treatment (Figure 4A). But the strongest effects were observed in the activity index with an increase of 142% (A3) and 116% (A7) after HT-treatment and 209% (A3) and 58% (A7) after HD-treatment (Figure 4C).

### 2.3. Benefits by HT and HD in α-Synuclein-Induced Parkinsonian Models

**Figure 5:**
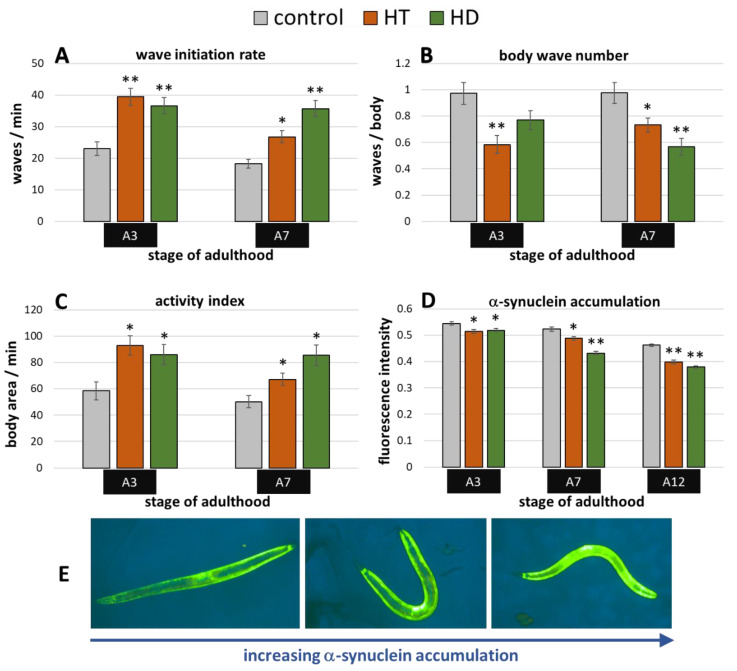
Benefits from HD & HT treatment in the OW13 strain. The nematode strain OW13 is characterized by α-synuclein expression in the body wall muscle cells. After polyphenol treatment, the wave initiation rate (**A**), the body wave number (**B**) and the activity index (**C**) were determined in two independent trials with ≥51 nematodes per treatment and age. Furthermore, the α-synuclein accumulation in muscle cells was quantified in two trials with ≥35 nematodes per treatment and age by fluorescence microscopy using a yellow filter (**D**). Data are presented as mean ± SEM. Differences compared to control were considered significant at *p* < 0.05 (*) and *p* < 0.001 (**). A3, A7, A12: 3rd, 7th and 12th day of adulthood. Finally, examples for the appearance of α-synuclein accumulation with increasing fluorescence intensities are shown (**E**).

The transgenic *C. elegans* synucleinopathies-model ‘OW13′ features α-synuclein expression in the body wall muscle cells driven by the muscle specific *unc-54*-promoter. The resulting movement deficits were already described by Van Ham et al. [51] and were also visible in the current study. The wave initiation rate, for instance, deteriorated by more than 50% when comparing untreated wild type animals (Figure 2A) with OW13 nematodes (Figure 5A) at the 3rd day of adulthood. Both polyphenols were able to mitigate the α-synuclein-induced locomotion impairments, whereas HD showed slightly higher capacities in aged (A7) nematodes (Figure 5A–C): At the 7th day of adulthood, the wave initiation rate (Figure 5A), the body wave number (Figure 5B), and the activity index (Figure 5C) were improved by HD-treatment by 96%, 42%, and 70%, respectively, whereas HT led to an enhancement by 47%, 25 %, and 34%, respectively.

The accumulation of α-synuclein in the muscle cells can be directly observed and quantified in the OW13-strain (Figure 5E). This is possible due to the transparent nature of *C. elegans* and due to the linkage of the yellow fluorescent protein (YFP) to the synthesized α-synuclein. Thus, fluorescence microscopy enabled the detection of potential α-synuclein-inhibiting abilities of the tested polyphenol treatments. Indeed, both treatments led to a reduction of accumulated YFP (Figure 5D), which is a direct indication for the decrease of the α-synuclein amount. The enhancement by the polyphenols is quite similar at A3 (5–6%), whereas HD showed clearly stronger effects at A7 and A12 with a decrease of 17% and 18%, respectively, compared to HT-treated nematodes with a decrease of 7% and 14% (Figure 5D). The overall reduction of the fluorescence intensities with age is based on the aging-dependent decline of *unc-54* expression [52].

**Figure 6:**
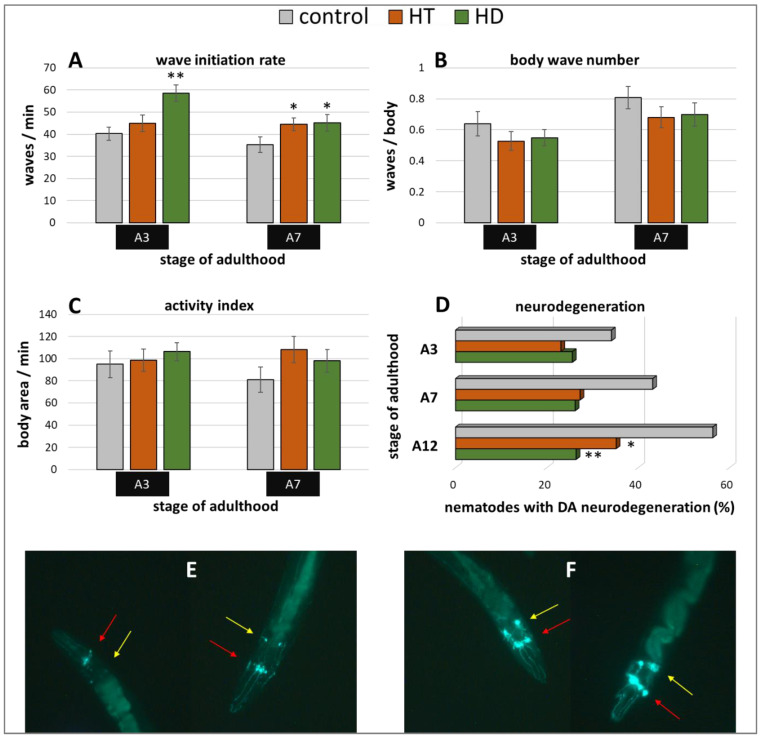
Benefits from HD & HT treatment in the UA44 strain. The nematode strain UA44 is characterized by α-synuclein as well as GFP expression in dopaminergic neurons. After polyphenol treatment, the wave initiation rate (**A**), the body wave number (**B**) and the activity index (**C**) were determined in two independent trials with ≥48 nematodes per treatment and age. Furthermore, the neuronal viability was analysed in three trials with ≥39 nematodes per treatment and age by fluorescence microscopy using a green filter. Shown are the percentages of nematodes with degenerated dopaminergic anterior neurons with and without polyphenolic treatment (**D**). Data are presented as mean ± SEM. Differences compared to control were considered significant at *p* < 0.05 (*) and *p* < 0.001 (**). A3, A7, A12: 3rd, 7th and 12th day of adulthood. Two nematodes with neurodegeneration characterized by missing or weak fluorescence in the DA neurons (**E**) as well as two nematodes with intact neuronal appearance (**F**) are shown. The DA neurons are sub-classified as four CEP neurons (red arrows), which are superimposed in most pictures and two ADE neurons (yellow arrows).

Furthermore, the Parkinson’s model ‘UA44′ was used to investigate the anti-Parkinsonian capacities of the polyphenol treatments. This strain is characterized by the expression of α-synuclein in dopaminergic neurons, which does not lead to distinct movement deficits [53] but to accelerated neurodegeneration [54]. Interestingly, only the wave initiation rate could profit from HT and HD treatment in this model (Figure 6A): HT increased the rate by 11% (A3) and 26% (A7) and HD by 45% (A3) and 28% (A7). No enhancement could be observed in the body wave number (Figure 6B) or the activity index (Figure 6C) by either polyphenol treatment.

In the UA44 strain, the green fluorescent protein (GFP) is linked to the dopamine transporter in dopaminergic neurons, thus, the vitality of the six anterior and two posterior dopaminergic (DA) neurons can be observed with a fluorescent microscope. The α-synuclein-induced damage of the nerve cells manifests as lowered or missing fluorescence in single neurons, whereas the classification of intact and degenerated anterior DA neurons were performed as described in Harrington et al. [55] and as illustrated in Figure 6E,F. The increase of degenerated neurons with age could be completely blocked by 250 µg/mL HD (Figure 6D). In all age classes, the quantity of degenerated anterior DA neurons constantly amounts to about 26% in the HD-treated group, whereas the quantity of degenerated neurons in the control group increased by 22% from 34% (A3) to 56% (A12). HT-treated nematodes also featured a neuroprotective effect, however, an increase of neurodegeneration with age is still visible.

### 2.4. Summary and Comparison of HD- and HT-Action in All Bioassays

**Figure 7:**
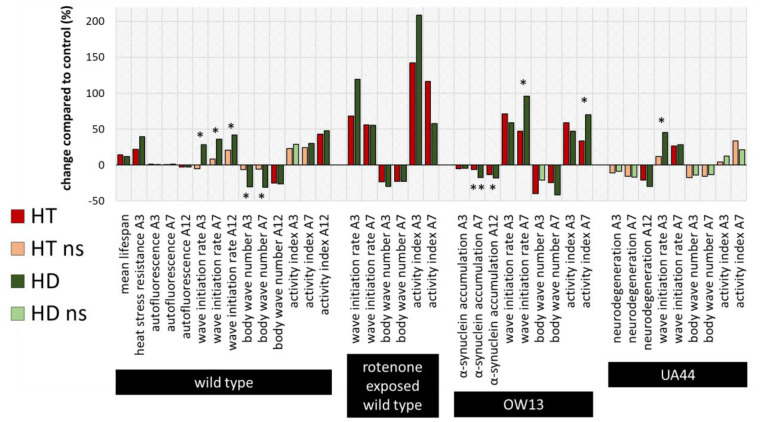
Summarized percentage changes after polyphenol treatment in all bioassays. The graph shows the percentage changes in all measured parameters compared to the respective control. Dark red (HT) and dark green (HD) bars represent significant (*p* < 0.05 or *p* < 0.001) and light green or red bars non-significant (ns) differences to the control. In addition, the differences between the HT- and HD-treated groups were statistically determined and the differences between the treated groups were considered significant at *p* < 0.05 (*) and *p* < 0.001 (**). Due to the distinct performance of the assays with temporal distance and separate control-groups, no direct comparison of HT and HD was conducted for the lifespan and heat stress resistance assy. A3, A7, A12: 3rd, 7th and 12th day of adulthood.

To compare the efficiencies of the action of HT and HD in all bioassays, the percentage changes relative to the respective control without polyphenol treatment were calculated and illustrated (Figure 7), whereas saturated and light-coloured bars represent significant and non-significant changes to the control, respectively. Furthermore, the measurements in the HT- and HD-treated groups were statistically compared with each other and labelled with * (*p* < 0.05) or ** (*p* < 0.001). Both polyphenol treatments are very efficient in enhancing all swim parameters in the rotenone PD-model and no significant differences could be detected between the HT- and HD-treated groups. However, in the wild type, the advantage of HD compared to HT is visible in two of the three locomotion characteristics. Since the measurements of lifespan and heat stress resistance were performed separately and with different control-groups, no direct comparison was possible in these cases. The effect of HD and HT was quite similar in the UA44 transgenic strain; only the wave initiation rate of young UA44-nematodes differed in HT- and HD-treated nematodes with a significant advantage in the HD-treated group. Moreover, the advantage of HD compared to HT is clearly visible in older OW13 nematodes, where α-synuclein accumulation, wave initiation rate and activity index featured a more pronounced benefit from the HD-treatment. None of the measurements in any strain exhibited an advantage of HT compared to HD.

## 3. Discussion

### 3.1. HD and HT Enhance Health and Longevity of Wild type C. elegans

Many studies reported the beneficial effects of polyphenols and flavonoids to improve health and extend lifespan in *C. elegans* [56,57]. Furthermore, healthy aging can be enhanced by several broad factors such as hormesis, autophagy and calorie restriction that increase stress resistance and longevity in *C. elegans* [58,59]. Healthspan is hard to define since it comprises the core features of health, that is physiological, cognitive, physical and reproductive function as well as a lack of disease [44]. To determine and compare the effects of treatments in terms of healthspan, it is necessary to operationalize features of health and thus, enable an objective way to measure healthspan. In this regard, the identification of predictive biomarkers and molecular pathways of health are mandatory to finally suggest applicable interventions, such as nutrition and exercise [44]. In the present study, we have demonstrated that the polyphenolic preparations HD and HT at a concentration of 250 µg/mL prolong lifespan and improve healthspan, determined via several physiological and functional parameters such as stress resistance, age pigment accumulation and swim behaviour in old wild type worms. The most powerful predictor of longevity and healthspan in old worms seems to be movement [60,61]. Similar to humans, the ability of *C*. *elegans* to move diminishes with aging [62]. Interestingly, HD also exerted optimal performances regarding the impact on locomotion in older wild type nematodes, which can be interpreted as an anti-ageing effect, while HT treatment did not show such evident improvements.

To treat *C. elegans* as naturally as possible, a live bacterial food was used throughout their life in this study. However, this protocol could also create some problems, which should be mentioned here. By adding these compounds to the living feeding bacteria, compound-bacteria interactions cannot be excluded. It is known that several plant polyphenols possess antibacterial activities [63]. These potential antimicrobial abilities could result in the inhibition of bacterial proliferation, which in turn would reduce the harmful intestinal accumulation of bacteria during ageing and prolong healthspan in *C. elegans* [64,65,66]. However, a recent study by Medina—Martínez et al. [67] showed, that at least 400 µg/mL HT were necessary to produce a perceptible growth deceleration of different *E. coli* strains. Furthermore, also olive leaf extract was shown to be only weakly active against *E. coli* bacteria [68]. On the other hand, it also cannot be excluded that compound-bacteria interactions lead to the degradation of the test compound by the bacteria. At least for hydroxytyrosol, this possibility can be neglected according to the study by Medina—Martínez et al. [67] who showed that degradation by *E. coli* was present to only a small extent. Nevertheless, in future studies the metabolic profile of *C. elegans* after the digestion of HT and HD in combination with alive *E. coli* should be checked for potential degradation products.

### 3.2. Hormesis Is Involved in Neuroprotective Effects from HD

Mild stress-induced hormesis represents a promising strategy to improve longevity and healthy aging. A recent paper demonstrated that hormesis leads to ageing-deceleration and highlighted its beneficial effects on lifespan, overall healthspan and especially stress resistance by activation of *daf-16*, *sod-3*, *ctl-1*, *hsp-16.2* and *sir-2.1* longevity genes in *C. elegans* [69]. Other recent studies also indicated the anti-aging effect of hormesis and considered it as an overcompensation stress response to the disruption in homeostasis via HSF-1 and SKN-1/Nrf2 signalling pathways [70,71]. The transcription factor SKN-1, the *C. elegans* orthologue of mammalian Nrf2 protein, is a well-known longevity factor that plays an essential role in oxidative stress response. It has also been reported that activation of SKN-1 induces the suppression of DAF-16, another well-known longevity gene, leading to detrimental effects on stress resistance and lifespan in *C. elegans*. In the same study, it has been demonstrated that oleic acid induces protective actions by regulating DAF-16 to promote lifespan extension and health in ageing worms [72]. In addition, a recent study reported that a chaperone complex mediates longevity response between HSF-1 and DAF-16/FOXO by reducing insulin/IGF-1 signaling to increase the lifespan in *C. elegans* [73]. The 90-kDa heat-shock protein (HSP-90) is an essential, evolutionarily conserved eukaryotic molecular chaperone [74]. Consistent with this concept, a recent study showed that DAF-21/HSP-90 is required for *C. elegans* longevity and provides a functional crosstalk between the proteostasis and nutrient signaling networks by ensuring DAF-16/FOXO isoform A activity [75]. Interestingly, the sirtuin family, named after mammalian Sirtuin 1 (SIRT1), features a high number of sirtuin-orthologs in several organisms, such as SIR-2.1 in *C. elegans*. Recently, it has been demonstrated that HSP-90/Hsp90 modulates lifespan via SIR-2.1/SIRT1 in *C. elegans* and in mammalian cells [76].

The Brunetti et al. [15] findings were supportive of a hormetic dose response for hydroxytyrosol with the optimal concentration at 250 µg/mL, the same concentration employed in the present study within a similar preconditioning experimental system. These findings are consistent with a substantial body of research that shows that preconditioning experiments which employed an adequate number of conditioning doses typically demonstrate a hormetic dose response [77,78], which is characterized by a low dose stimulation with the maximum response typically about 30–60% greater than the control response, similar to what was reported here. Thus, a hormetic background mechanisms for the action of HD seems plausible. Hidrox^®^ has an excellent safety profile, with no adverse effects even at a very high dose [41]. Consistent with this notion, in vitro studies have evaluated HT as a non-genotoxic and non-mutagenic compound with NOAEL (No Observed Adverse Effects Level) classification, suitable for long-term consumption [79]. Thus, the safety profile of Hidrox^®^ makes it an excellent food supplement.

### 3.3. Implications in PD Pathogenesis

Delaying aging, e.g., as seen after HD treatment, is a neuroprotective mechanism that may provide potential prevention in worm models of PD [80]. Parkinson’s disease (PD) is a neurodegenerative disorder characterized by a severe depletion in number of dopaminergic cells of the *substantia nigra* (SN). As an effect of this reduction in dopaminergic neurons, a significant fall in brain dopamine (DA) levels occurs [81]. Although several hypotheses have been raised, including (i) defective DNA repair mechanisms, (ii) specific genetic defects, (iii) mitochondrial dysfunction, or (iv) toxic compounds in the environment, none of these, alone, completely explains the cascade of events responsible for the onset and progression of PD [81,82,83,84]. A large body of evidence demonstrates that free radicals play a key role in the pathogenesis of PD. In fact, there is a 10-fold increase in hydroperoxide levels in SN in PD, and dopaminergic neurons produce hydrogen peroxide either enzymatically, through the activity of mono-amine oxidase-A (MAO-A), or non-enzymatically via the intracellular autoxidation of DA [81,85]. Once formed, hydrogen peroxide by reacting with the reduced form of transition metals, such as Fe (II) and Cu(I), gives rise to the powerful oxidant hydroxyl radical and oxidative damage to nigral membrane lipids, proteins, and DNA ensues [86]. Reduced glutathione (GSH) significantly contributes to the detoxification of hydroxyl radical; in fact it reacts with the free thiol group of GSH which is oxidized to GSSG [86,87]. Unfortunately, SN has very low levels of GSH compared with other brain areas and this contributes to the triggering of PD pathogenesis by free radicals [88].

A number of worm models of PD have been generated through either exposing worms to a neurotoxic chemical such as 1-methyl-4-phenylpyridium ions (MPP+) or *6*-*hydroxydopamine* (6-OHDA) or by reproducing the genetic defect present in the inherited form of PD [53]. In particular, many recent data suggest a key role played by phenolic components of extra virgin olive oil (EVOO) in counteracting protein misfolding and proteotoxicity, with a particular emphasis on the mechanisms leading to the onset and progression of PD. Notably, HT efficiently neutralizes free radicals and protects biomolecules from ROS-induced oxidative damage. In this regard, HT activates the Nrf2–antioxidant response element (ARE) pathway, leading to the activation of phase II detoxifying enzymes and the protection of dopaminergic neurons exposed to hydrogen peroxide or to 6-hydroxydopamine [89,90,91]. These protective enzymes include NADPH quinone oxidoreductase-1, heme oxygenase-1, glutathione S-transferase, and the modifier subunit of glutamate cysteine ligase, which catalyzes the first step in the synthesis of GSH [10]. It is noteworthy that HT, a product of dopamine metabolism, is present in the brain [92]. Specifically, monoamine oxidase (MAO) catalyzes oxidative deamination of dopamine in a neurotoxic metabolite, DOPAL (3,4-dihydroxyphenylaldehyde) in dopaminergic neurons [93]. The latter can be oxidized by aldehyde dehydrogenase (ALDH) to DOPAC (3,4-dihydroxyphenylacetic acid), the major metabolite of dopamine in the brain or may be reduced to HT by alcohol dehydrogenase (ADH). At the same time, DOPAC reductase can transform DOPAC into HT [94]. DOPAL is a highly reactive metabolite, suggesting that it might be a neurotoxic dopamine metabolite with a role in the pathogenesis of PD.

Several studies reported the neurotoxicity of DOPAL in vivo and strongly suggest its role in PD pathogenesis [95].

The health effects of olive polyphenols, particularly HD and HT on the ageing process in old worms (i.e., increased locomotion and reduced intestinal autofluorescence) have been reported here. Our study reinforces the hypothesis that HD is protective against PD pathogenesis. This is in agreement with other studies showing that the olive fruit derivatives oleuropein and HT, as well as other polyphenols, such as the green tea derivative epigallocatechin 3-gallate (EGCG) inhibit DA-related toxicity and protect against environmental or genetic factors that induce DA neuron degeneration by the modulation of Nrf2-Keap1 and PGC-1α anti-oxidative signaling pathways in vitro and in vivo [96,97,98]. In addition, another recent study suggested that tyrosol from EVOO is effective in reducing α-synuclein inclusions, resulting in a lower toxicity and extended lifespan of treated transgenic nematodes [99].

Notably, α-synuclein is an aggregation-prone neuronal protein whose cellular function is not well known. As *C. elegans* has no orthologue of this protein, worm models have been generated by overexpression of wild-type or mutant forms of human α-synuclein in different tissues (i.e., either body-wall muscle, pan-neuronal, or only in the dopaminergic neurons). In most cases, overexpression leads to locomotion defects or the degeneration of dopaminergic neurons [26].

Interestingly, both polyphenols mitigated, age-dependently, the build-up of human α-synuclein in the body wall muscle cells of a transgenic *C. elegans* model (strain OW13) and improved swim performance. Our results are relevant to PD pathogenesis, due to the central role of mitochondria in metabolism, ROS regulation, and proteostasis [100]. The extent to which these pathways, including the mitochondrial unfolded protein response (UPR) and mitophagy, are active may predict severity and progression of these disorders, as well as sensitivity to chemical stressors. This holds true when considering, in a PD-like context, that transgenic nematodes express the Lewy body constituent protein α-synuclein. In fact, recent studies suggested that co-expression of α-synuclein and ATFS-1-associated dysregulation of the UPR synergistically potentiate dopaminergic neurotoxicity [101]. Moreover, other evidences have demonstrated that, in *C. elegans*, the inducible transcription factor SKN-1, directly controls UPR signaling and controls the transcription factor genes of XBP-1 and ATF-6 [102].

### 3.4. HD Is More Effective in Health Maintenance than Pure HT

In the present paper, we compared the health effects of Hidrox^®^ and hydroxytyrosol as neuroprotective agents in *C. elegans* wild type and PD models. An important consideration that has emerged from this study relates to the different strength of biological activities delivered by hydroxytyrosol in its “natural” environment versus hydroxytyrosol in its purified, synthetic form. Although HT and HD were formulated to the same concentrations of 250 µg/mL, the two formulations used in the experiments contain dramatically differing hydroxytyrosol concentrations. HD as a raw formulation, indeed, contains a much smaller amount of hydroxytyrosol. 250 µg of HD correspond to 30 µg total polyphenols and only approximately 15 µg hydroxytyrosol. Thus, in comparison to the HT formulation (100% hydroxytyrosol), HD has 1/17th of the hydroxytyrosol in HT, but provides similar or even higher activity in all the assays so far described.

The difference between a “natural” formulation of olive polyphenols and purified fractions of olive polyphenols is, however, not surprising. Several studies published in the literature [14,21,42] have confirmed that hydroxytyrosol, stripped from its natural environment and/ or cofactors which can be in minute concentrations in the juice of olives, provided a much less and limited range of activities in vitro and in vivo. Therefore, HD activity cannot just be accounted by and attributed solely to hydroxytyrosol. While this study confirms that “natural” HD has a superior range of activities than its purified or synthetic hydroxytyrosol counterpart, further studies are needed to support this conclusion.

Due to its composition (see chromatogram in Figure S1), it is plausible that other polyphenols than HT alone, might be involved in the beneficial action of HD. HD, in fact, contains compounds such as oleuropein, oleuropein aglycone, tyrosol, and gallic acid, to only name a few [41,103]. Previous studies already showed their abilities to improve the life- and healthspan of the nematodes [15,35,40,98], thus, it could be argued that the beneficial action of HD is due to one of those polyphenolic ingredients independent of HT. However, oleuropein, for instance, being the second most abundant polyphenol in HD, is only present in trace amounts. Only about 1% of HD consists of oleuropein so that the final concentration of 250 µg/mL HD delivers only about 2.5 µg/mL oleuropein. In our previous study, we could show that even 30 µg/mL HT or oleuropein aglycone were not sufficient to provide healthspan benefits [15]. Thus, it is unlikely that any single polyphenol included in HD is responsible for the observed effects. More likely, the polyphenols act in concert, whereas HT as the most abundant polyphenol in HD plays a central role. Additional experiments with chemically defined mixtures of polyphenols and other HD ingredients must be performed in future to find the responsible elements for healthspan enhancement by HD.

Our findings are in agreement with other studies indicating a higher cytoprotective activity of HD than that of purified HT. Consistent with this line of evidence, in vitro studies have measured the damage produced to the cell membrane by potent oxidant agents like H_2_O_2_ and 15-HPETE in bovine heart endothelial cells, and revealed that HD was extremely active in preventing membrane damage even at concentrations in micromolar range (10^−6^), while pure HT obtained by HPLC separation was not protective against oxidant insult and damage [104]. On the contrary, high concentrations of purified HT produced a pro-oxidation effect and increased the cytotoxicity of the oxidants [103]. Another in vitro study confirmed that HD had higher antioxidant and anti-inflammatory activity than the same amount of pure HT, as demonstrated following the oxidation of mitochondrial membrane lipids by free radicals applying Electron Spin Resonance (ESR) spectroscopy (using Superoxide, HO radical and NO radicals as toxic agents) [104]. Furthermore, by measuring the effect of HT and HD on atherosclerosis lesions in an animal model, it was shown that the phenolic-enriched olive product (HT), out of its original matrix, could not only be not beneficial but actually harmful. The results suggest that the formulation of functional foods may require maintaining the natural environment in which these molecules are found [105]. In humans, HD administrated orally (1 mL of Olivenol, 2.5 mg HT) significantly increased plasma antioxidant activity and, in addition, bioavailability studies have established that, after ingestion, the absorption of HT from olive oil is dose-dependent, with low adsorption bioavailability after administration of a high amount of pure HT [106].

## 4. Materials and Methods

### 4.1. C. elegans Maintenance

The wild type *C. elegans* strain N2 (var. Bristol), the transgenic *C. elegans* strain OW13 (grk-1(ok1239); pkIs2386 [unc-54p::α-synuclein::YFP + unc-119(+)]) as well as the *Escherichia coli* feeding strain OP50 were obtained from the Caenorhabditis Genetics Centre (CGC, University of Minnesota, Minneapolis, MN, USA). The *C. elegans* strain UA44 (baIn11[Pdat-1::α-synuclein, Pdat-1::GFP]) was kindly provided by the Caldwell laboratory (University of Alabama, Tuscaloosa, AL, USA) [107]. All nematodes were maintained on standard nematode growth medium (NGM) agar plates at 22 °C, seeded with OP50 according to Brenner [108]. Prior to all tests, synchronized *C. elegans* populations were obtained by dissolving young adults in a 3% sodium hypochlorite solution according to Stiernagle [109]. The obtained eggs hatched in M9 buffer overnight, and were transferred to new NGM plates the following day. About 48 h later, L4 larvae were transferred to treatment and control plates. In order to inhibit the reproduction, 100 µM 5-fluoro-2-deoxyuridine (FUdR) was added to each plate [110].

### 4.2. Polyphenol and Rotenone Treatment

The treatment plates were prepared with the polyphenol hydroxytyrosol (HT; Sigma-Aldrich, St. Louis, MO, USA or with the aqueous olive pulp extract “HIDROX^®^” (HD). The HD extract was provided by Oliphenol LLC (Hayward, CA, USA), and total polyphenolic content of the HD extract was declared as 12% [111]. Among the polyphenolics, the major constituent of HD is hydroxytyrosol (40–50%), while other polyphenols present include oleuropein (5–10%), tyrosol (0.3%), and about 20% of other polyphenols including oleuropein aglycone and gallic acid [41]. HD is a freeze-dried powder prepared from the acidic hydrolysis (citric acid 1%) of the aqueous fraction of olives extracted from the defatted olive pulp, a byproduct during the processing of olives (*Olea europaea* L.) for olive oil extraction [41]. Hidrox^®^ is titrated on the total content of olive polyphenols (12%).

HT and HD were dissolved in bidistilled water at 60 mg/mL and the solutions were stored at −20 °C. HT or HD, respectively, were added to the bacteria and agar at a final concentration of 100–500 µg/mL.

To trigger the Parkinsonian phenotype, wild type nematodes were exposed to rotenone. A stock solution of 0.5 mg/mL rotenone was prepared in DMSO and added to a final concentration of 10 µM to the control and polyphenol plates. After distribution with a spatula and drying for 24 h in the dark, OP50 (including 10 µM rotenone and the respective polyphenol) was spread on the plates. L4 nematodes were transferred to the rotenone plates until they were used for bioassays.

### 4.3. Lifespan and Heat Stress Assay

Synchronized wild type nematodes were observed with a stereo microscope and scored for their survival by gently touching them with a platinum wire. The animals were counted daily from the first day of adulthood until all died. Contaminated plates as well as vanished nematodes were censored. The heat stress test was performed according to the lifespan protocol, however, nematodes were stressed by heat (37 °C) for 3 h at the third day of adulthood and counting of dead and alive worms started 1 day after stress exposure.

### 4.4. Fluorescence Microscopy Analysis

For the fluorescence observation, several nematodes were placed on a 2% agar pad on a microscope slide and anesthetized with 4 µL NaN_3_ (1M). The images were taken with the aid of the Axioskop fluorescence microscope (Carl Zeiss, Oberkochen, Germany) and filter sets from the Zeiss 4880 series.

To determine the autofluorescence in wild type nematodes, the images were captured at 100× magnification with a red filter set (TRITC, 545/30 nm ex, 610/70 nm em). In addition, bright field images were used to define the shape of each worm. The CellProfiler Software [112,113] was used to determine the mean red fluorescence intensity per total worm body.

The OW13 transgenic strain features yellow fluorescent protein linked to α-synuclein in the body wall muscle cells. Therefore, the nematodes were captured by using a yellow barrier filter with 100× magnification. The images were processed using the CellProfiler software and the yellow fluorescence intensity emitted per total worm body was calculated.

The UA44 transgenic strain features GFP linked to the dopamine transporter in the six dopaminergic neurons of the head and two in the tail as well as α-synuclein expression in dopaminergic neurons. To analyse the vitality of the neurons, the green barrier filter was used with a 200× magnification. The number of detectable anterior neurons were finally counted and assayed for patterns of degeneration, as described previously from Harrington et al. [55].

### 4.5. Swim Behaviour Assay

Wells with a depth of 0.5 mm and Ø 10 mm were created with two self-adhesive marking films for microscope slides and filled with M9 buffer. 5 to 10 nematodes were transferred per well, covered by a cover slip, and recorded for 60 s with a connected camera. Each video was converted into single frames which were analysed with the CeleST software as described by Restif et al. [47] and Ibáñez-Ventoso et al. [48]. The wave initiation rate, the body wave number and the activity index were evaluated as representative parameters of locomotive behaviour.

### 4.6. Statistical Analysis

All experiments were independently conducted at least two times. The Online Application for Survival Analysis (OASIS 2; https://sbi.postech.ac.kr/oasis2/) [114] was used for comparing survival differences between two conditions. Fluorescence intensities as well as swim behaviour parameters were calculated as mean ± SEM and statistical significance was calculated by two tailed t-test using GraphPad (https://www.graphpad.com/quickcalcs/). Chi-square test was used to compare the number of worms with intact and degenerated neurons in the UA44 strain.

## 5. Conclusions

Both polyphenol treatments—pure hydroxytyrosol and the natural preparation ‘Hidrox^®^’—were able to similarly improve the lifespan, stress resistance as well as age pigment accumulation in wild type nematodes. Furthermore, the beneficial locomotion effects of HD and HT are quite equally strong in the rotenone-stressed PD-model of *C. elegans*. However, the abilities of HD and HT also provide some differences: HD exposure led to much stronger beneficial locomotion effects in wild type worms compared to HT. Moreover, also the PD-model characterized by α-synuclein expression in muscles (strain OW13) did benefit significantly more from HD than HT. Only in the UA44 strain, which features α-synuclein expression in DA-neurons, the beneficial effects of HD and HT are rather weak with only one minor advantage of HD over HT. Thus, the hypothesis that HD features higher healthspan-promoting abilities than HT was only partly confirmed. Further studies are needed to uncover the molecular background mechanisms which led to this distribution of effects. Nevertheless, both polyphenolic treatments have the potential to partly prevent or even treat ageing-related neurodegenerative diseases and ageing itself. Future investigations including mammalian models and human clinical trials are needed to uncover the full potential of these olive ingredients.

## Supplementary Materials

Supplementary materials can be found at https://www.mdpi.com/1422-0067/21/11/3893/s1.

## Abbreviations

ADH: Alcohol dehydrogenase; ALDH: Aldehyde dehydrogenase; ARE: Antioxidant response element; C. *elegans*: Caenorhabditis elegans; CGC: Caenorhabditis Genetics Centre; DA: Dopaminergic neurons; DOPAC: 3,4-dihydroxyphenylacetic acid; DOPAL: 3,4-dihydroxyphenylaldehyde; EGCG: Epigallocatechin 3-gallate; ESR: Electron Spin Resonance; EVOO: Extra virgin olive oil; GFP: Green fluorescent protein; GSH: Reduced glutathione: GSSG: Oxidized glutathione; HD: Hidrox^®^/Olivenol Plus™; HT: Hydroxytyrosol; HSP-90: Heat-shock protein-90; MAO-A: Mono-amine oxidase-A; MD: Mediterranean diet; MPP+: 1-methyl-4-phenylpyridium ions; NGM: Nematode growth medium; 6-OHDA: 6-hydroxydopamine; OLE: Oleuropein aglycone; OVW: Olive vegetation water; PD: Parkinson’s disease; SN: Substantia nigra; SNpc: Substantia nigra pars compacta: SIRT1: Sirtuin 1; UPR: Unfolded protein response; YFP: Yellow fluorescent protein
