# Supplementary material for: Healthspan Enhancement by Olive Polyphenols in C. elegans Wild Type and Parkinson’s Models
Source: Int J Mol Sci. 2020 May 29;21(11):3893. doi: 10.3390/ijms21113893 (PMC7312680; doi:10.3390/ijms21113893)

**Table S1: Heat-stress survival characteristics during HD and HT treatment**

| treatment | n | mean survival (days) | SEM | days until deaths of population reached | | | | |
|---|---|---|---|---|---|---|---|---|
| | | | | 25 % | 50 % | 75 % | 90 % | 100 % |
| **control (water)** | 114 | 2.96 | 0.07 | 1.95 | 2.44 | 2.91 | 3.58 | 5 |
| **100 µg/ml HD** | 66 | 3.35* | 0.12 | 2.15** | 2.83** | 3.5 | 3.9 | 8 |
| **250 µg/ml HD** | 62 | 4.13** | 0.21 | 2.38** | 3.33** | 4.69** | 5.85** | 9 |
| **500 µg/ml HD** | 68 | 3.99** | 0.17 | 2.38** | 3.39** | 4.55** | 5.52** | 7 |
| | | | | | | | | |
| **control (water)** | 90 | 2.23 | 0.04 | 1.33 | 1.65 | 1.98 | 2.57 | 3 |
| **100 µg/ml HT** | 52 | 2.48* | 0.08 | 1.45* | 1.9* | 2.48 | 2.85 | 4 |
| **250 µg/ml HT** | 65 | 2.71** | 0.07 | 1.71** | 2.25** | 2.68* | 2.93* | 4 |
| **500 µg/ml HT** | 79 | 2.54** | 0.07 | 1.49** | 1.99** | 2.55* | 2.89* | 4 |

Differences compared to control were considered significant at p <0.05 (*) and p <0.001 (**). p-value determination was realized with Log-Rank Test for the mean lifespan and Fisher's Exact Test for specific time points.

**Figure S1.** GC-MS analysis and phenolic compound identification, by spectrum matching and library searching in the NIST/EPS/NIH Mass Spectral Library Database, of of HD. Peaks: 1, tyrosol; 2, Vanilic acid; 3, Hydroxytyrosol; 4, 3,4-dihydroxybenzoic acid; 5, citric acid (hydrolysis acid to obtain HD from OVW, olive acqueous vegetation water); 6, syringic acid; 7, gallic acid; 8, caffeic acid (trace); 9, 3-hydroxy,4-methoxyphenylacetic acid; 10, gentisic acid (trace).

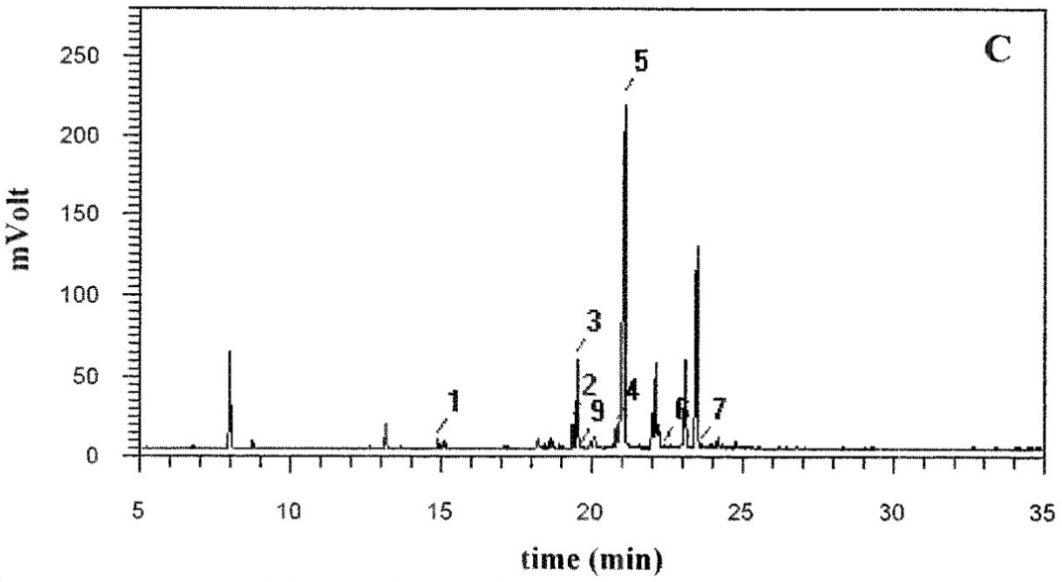

Supplement: Supplementary file 1 [file ijms-21-03893-s001.pdf]
